# The Impact of the Endocrine and Immunological Function of Adipose Tissue on Reproduction in Women with Obesity

**DOI:** 10.3390/ijms25179391

**Published:** 2024-08-29

**Authors:** Katarzyna Mączka, Olga Stasiak, Paulina Przybysz, Monika Grymowicz, Roman Smolarczyk

**Affiliations:** 1 Department of Gynecological Endocrinology, Medical University of Warsaw, 00-315 Warsaw, Poland.; katarzyna.maczka@wum.edu.pl (K.M.);; 2 Doctoral School, Medical University of Warsaw, 02-091 Warsaw, Poland

**Keywords:** obesity, adipokines, leptin, adiponectin, resistin, kisspeptin, inflammation, hypothalamus, reproduction, fertility

## Abstract

Obesity, which leads to metabolic dysregulation and body function impairment, emerges as one of the pressing health challenges worldwide. Excessive body fat deposits comprise a dynamic and biologically active organ possessing its own endocrine function. One of the mechanisms underlying the pathophysiology of obesity is low-grade systemic inflammation mediated by pro-inflammatory factors such as free fatty acids, lipopolysaccharides, adipokines (including leptin, resistin and visfatin) and cytokines (TNF-α, IL-1β, Il-6), which are secreted by adipose tissue. Together with obesity-induced insulin resistance and hyperandrogenism, the exacerbated immune response has a negative impact on the hypothalamic–pituitary–gonadal axis at all levels and directly affects reproduction. In women, it results in disrupted ovarian function, irregular menstrual cycles and anovulation, contributing to infertility. This review focuses on the abnormal intracellular communication, altered gene expression and signaling pathways activated in obesity, underscoring its multifactorial character and consequences at a molecular level. Extensive presentation of the complex interplay between adipokines, cytokines, immune cells and neurons may serve as a foundation for future studies in search of potential sites for more targeted treatment of reproductive disorders related to obesity.

## 1. Introduction

Obesity is a complex disease defined as the accumulation of excessive adipose deposits or their abnormal distribution. According to the World Health Organization (WHO), the cut-off point for diagnosing obesity is a body mass index (BMI) equal or higher than 30 kg/m^2^ [1]. This morbid condition has a multifactorial background and poses a great threat to human health. By significantly increasing the risk of non-communicable diseases, obesity has been globally recognized as a major determinant of disability and death and has already reached pandemic scale [1,2].

Based on the morphology and different roles in maintaining energy homeostasis, two distinct types of adipose tissue (AT) can be distinguished—brown (BAT) and white adipose tissue (WAT). Furthermore, WAT is classified into visceral (VAT) and subcutaneous (SAT). The localization of VAT includes omental, mesenteric, retroperitoneal, gonadal and pericardial depots. Despite the site, it also varies from SAT in terms of the biochemical features and response to metabolic signaling [3,4]. An increased amount of visceral adipocytes has a greater impact on metabolic dysregulation than subcutaneous adipocytes due to the higher lipid synthesis and lipolysis rates. Moreover, VAT is closely related to portal circulation and its mediators can directly impair liver function [3]. Numerous studies have demonstrated a closer association between central obesity and obesity-related outcomes compared to general obesity, indicating that the waist–hip ratio (WHR) is a better predictor of risk associated with abundant AT than the BMI [5,6,7,8].

Obesity is inseparably associated with the presence of low-grade systemic inflammation. Besides serving as a storage compartment, WAT is an important, highly dynamic endocrine organ regulating energy expenditure and adjusting its responses to the metabolic demands of the organism. It is composed of a network of cellular elements, including mature adipocytes, pre-adipocytes, fibroblasts, endothelial cells and immune cells such as macrophages, neutrophils, eosinophils, mast cells, and T and B cells [9,10]. Therefore, to integrate all the components of the adipose stromal vascular fraction (SVF) and provide cross-talk between WAT and other organs, adipocytes and local immune cells produce a variety of biologically active compounds, which can be appropriately assigned as pro-inflammatory—leptin, resistin, visfatin, chemerin, progranulin, tumor necrosis factor α (TNF-α), dipeptidyl peptidase 4 (DPP4), retinol binding protein 4 (RBP4), WNT1-inducible signaling pathway protein 1 (WISP1), fatty acid-binding protein 4 (FABP4), plasminogen activator inhibitor-1 (PAI-1), follistatin-like 1 (FSTL1), monocyte chemoattractant protein–1 (MCP-1), secreted protein acidic and rich in cysteine (SPARC), SPARC-like protein 1 (SPARCL1), serum amyloid A (SAA), interleukin 6 (IL-6), interleukin 8 or CXC motif chemokine ligand 8 (CXCL8)—or anti-inflammatory—adiponectin, omentin, vaspin, isthmin 1, nesfatin 1, zinc-α2-glycoprotein (ZAG), secreted frizzled-related protein 5 (SFRP5), C1q/TNF-related protein 3 (CTRP3), lipocalin-2 (LCN2), interleukin 1 receptor antagonist (IL-1RA), and interleukin 10 (IL-10) [11,12]. Abnormal lipid aggregation, observed in obesity, leads to enhanced cell turnover and adipocyte hypertrophy, promoting pro-inflammatory agents and reactive oxygen species (ROS) secretion. Free fatty acids (FFA), a product of AT lipolysis, can directly bind to toll-like receptor 4 and 2 (TLR4 and TLR2), activating nuclear factor κB (NF-κB) and C-jun n-terminal kinase (JNK1) pathways, which stimulate adipocytes to release MCP-1 [13,14,15]. As a consequence, there is an increased macrophage infiltration into adipose tissue, accompanied by a propensity to convert from an anti-inflammatory M2 subtype to a pro-inflammatory M1 phenotype [4,12]. Responsible for initiating a sequence of protective mechanisms characterized by type 1 immune response, macrophages release an array of cytokines (including TNF-α, IL-6, and IL-1β), adipokines as well as nitrogen and oxygen intermediates [12,14,16,17]. Moreover, AT expansion, followed by underperfusion and hypoxia, activates the hypoxia-inducible factor 1 (HIF1) gene program in adipocytes, further advancing inflammatory processes. Mechanical stress accompanying cell enlargement and its impact on the extracellular matrix leads to collagen turnover disorders, additionally impairing lipid storage and energy metabolism [14]. Together, all these processes initiate a chronic response in adipose tissue, which directly contributes to the development of low-grade systemic inflammation. By promoting fibrosis, angiogenesis, neurodegeneration and cell necrosis, this condition is considered one of the underlying mechanisms for the development of metabolic and inflammatory diseases associated with obesity [10,11]. Circulating immune cells, cytokines and adipokines directly affect the gonads and structures of the central nervous system (CNS), integrating metabolic information with management of primary body functions.

The hypothalamus, acting as a supreme center of regulation of appetite and basal energy expenditure, is sensitive to peripheral signaling coming from gastrointestinal tract and AT, providing bidirectional communication between other brain structures as well as distant endocrine systems [18]. Evolutionarily, maintaining energy homeostasis has been crucial to ensuring successful reproduction, which is one of the fundamental physiological processes. The disruption of reproductive function in obesity is mainly caused by the endocrine imbalance, primarily manifested in altered insulin and adipokine profiles, resulting from the biochemical function of excessive AT [19]. Hormonal and immunological mediators have a direct impact on the hypothalamic–pituitary–gonadal (HPG) axis, gonadal receptors and peripheral androgen to estrogen conversion [17,19,20]. As a result, by affecting gonadotropin secretion, ovarian function and endometrial receptivity, this altered metabolic state contributes to menstrual disorders, delayed conceptions, increased miscarriage incidence and frequent failures in assisted reproduction treatment (ART) in women with obesity [20].

This review aimed to provide a comprehensive analysis of endocrine and immunological factors associated with adipose tissue, which contribute to menstrual cycle irregularity and impair fertility in women with obesity. By unravelling the molecular mechanisms connecting obesity with reproductive system disorders, we intended to explain the pathogenesis of infertility and highlight the impact of adipocyte function on general homeostasis.

From May to June 2024 a comprehensive search of major databases, including PubMed, Embase, ScienceDirect and ResearchGate, was conducted to find studies addressing the topic of the endocrine and immunological function of adipose tissue and its impact on reproduction, especially in females. The search strategy was based on specific MeSH terms, including adipocytes, adipose tissue, adipokines, leptin, adiponectin, resistin, obesity, kisspeptins, inflammation, hypothalamus, pituitary gland, ovary, reproduction, fertility and polycystic ovary syndrome, which were used alone or in combination.

## 2. Adipokines

**Figure 1:**
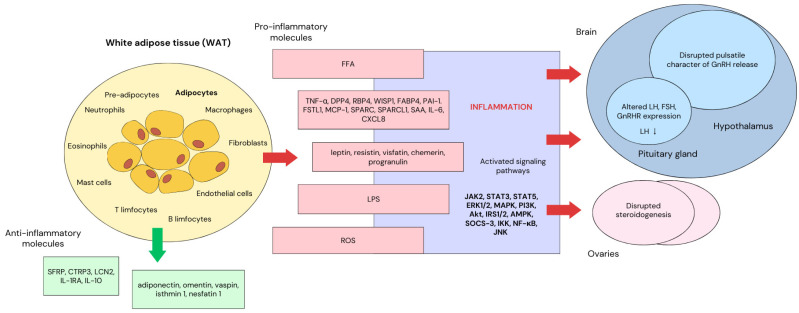
The impact of the secretory function of white adipose tissue on the HPG axis.

The adipose tissue is a metabolically active organ acting as the largest endocrine tissue in humans. Its pleiotropic function manifests through endocrine, paracrine, autocrine and nervous signaling, as well as immunological mechanisms. It secretes a great number of bioactive compounds, such as hormones, growth factors, enzymes, cytokines, complement factors and matrix proteins [21,22]. Adipokines, acting like classical hormones, can be divided into adipose-specific cytokines directly released by adipocytes (leptin, adiponectin, resistin, visfatin, omentin) and non-adipose-specific cytokines (RBP4, LCN2, chemerin, IL6, IL1β, TNFα) secreted by different cell types [20]. IL6, IL1β and TNFα are produced by macrophages residing in the AT [23]: RBP4, by adipocytes and, predominantly, hepatocytes [11,24], and LCN2, while chemerin, by both macrophages and neutrophils [25,26]. These products of AT secretion modulate a complex network of intracellular signal transduction and influence various physiological processes, such as energy and appetite modulation, thermogenesis, insulin sensitivity, hematopoiesis, osteogenesis, chondrogenesis, angiogenesis, blood pressure, atherosclerosis, neuroendocrine and immune response [12,20,21]. Moreover, through HPG axis disruption and altering ovarian physiology, they have a great impact on reproductive function [19] (Figure 1). In this review, we would like to focus mainly on adipokines produced by adipocytes and their effect on menstrual disorders and fertility.

### 2.1. Leptin

Leptin was one of the first identified adipokines and is well known for its pro-inflammatory properties and providing central weight control. It is considered an anti-obesity hormone as it promotes energy expenditure and suppresses appetite, reducing food intake. Obesity is closely associated with hyperleptinemia and leptin resistance. Leptin overexpression in the AT contributes to leptin receptor downregulation in target cells, which disturbs further intracellular signaling and decreases cell sensitivity to leptin [27].

This key adipocyte-derived hormone is a small peptide (16 kDa) consisting of 167 amino acids, which is encoded by the *obese* (*OB*) gene in mice or the *Leptin* (*Lep*) gene in humans [22,28]. The main site of leptin production is WAT, with a predominance of SAT. However, it has also been found in other organs, such as the skeletal muscles, the placenta, the hypothalamus, the pituitary and mammary glands, the intestines, and possibly, the ovaries [20,22,28,29,30]. Leptin belongs to the IL-6 family of cytokines and binds to the leptin receptor (LEPR), revealing its pleiotropic character affecting various tissues. LEPR is a member of the class I cytokine receptor superfamily and exists in six isoforms (a, b, c, d, e, f) as an effect of alternative RNA splicing [20]. Leptin receptors have been found in various structures of the CNS (hypothalamus, hippocampus, brain stem, cerebral cortex, midbrain) [31] and are especially highly expressed in the choroid plexus [20]. They are also present in peripheral organs such as the liver, thyroid, heart and pancreas [31]. LEPR isoforms can be classified into long (LEPRb), short (LEPRa, LEPRc, LEPRd, LEPRe and LEPRf) and secretory (LEPRe), which is the only soluble form [32]. Leptin is released into the bloodstream and serves as a bridge between the CNS and peripheral tissue, conveying information about energy status through interaction with LEPRb in the brain [22,33]. Some studies suggest that LEPRa is involved in leptin transfer across the blood–brain barrier (BBB) [34,35], allowing leptin to interact with LEPRb-expressing neurons in the hypothalamus and extrahypothalamic sites [35,36,37]. Together with LEPRa, other short isoforms (c, d, f) presumably also play an important part in the transportation of leptin to the CNS, its internalization and its degradation [31,32]. On the other hand, LEPRe binds leptin and regulates circulating leptin concentrations [31]. LEPRb is the only isoform containing a full-length intracellular domain, thus being able to fully activate the Janus kinase 2 (JAK2) as well as the signal transducer and activator of transcription proteins (STAT3 and STAT5) pathways [12,29,38]. The JAK2-STAT3/5 pathway is the most important signaling mechanism initiated by leptin. While activation of STAT3 signaling suppresses feeding, STAT5 modulates endocrine function and leptin’s effect on reproduction [39]. Moreover, LEPRb- binding activates other pathways involving mitogen-activated protein kinases (MAPKs), which include extracellular signal-regulated kinase 1/2 (ERK 1/2), insulin receptor substrate (IRS), phosphatidylinositol 3-kinase (PI3K), protein kinase B (Akt) and 5′ adenosine monophosphate-activated protein kinase (AMPK) [40,41]. These alternative pathways are responsible for maintaining homeostasis by regulating fundamental physiological processes, such as apoptosis, cell proliferation, differentiation, growth and autophagy [31]. In addition to this, leptin-induced activation of IRS-PI3K-Akt acts synergistically to classic activation of the JAK2-STAT3/5 pathway, reducing food intake and increasing energy expenditure [31,33].

Leptin has an impact on all the components of the HPG axis. It demonstrates stimulatory effects in the CNS and, consequently, indirectly modulates ovarian function through GnRH. However, at the level of the ovary itself, it shows direct suppressive action by antagonizing the effects of growth factors and inhibiting steroidogenesis [29]. The superior regulator of the reproductive axis is the gonadotropin hormone-releasing hormone (GnRH) secreted by GnRH neurons located mainly in the preoptic area of the hypothalamus. Although leptin stimulates GnRH secretion, GnRH neurons do not show LEPRb expression [42]. The effect of leptin is mediated through other neuropeptides, including proopiomelanocortin (POMC), neuropeptide Y (NPY), Agouti-related peptide (AgRP), γ-aminobutyric acid (GABA) and kisspeptin, which will be separately discussed later in this review [22,29,39]. The hypothalamic signaling pathways triggered by leptin start in the ventral premammillary nucleus (PMV) and arcuate nucleus (ARC), stimulating kisspeptin expression and inhibiting the orexigenic AgRP–NPY–GABAergic neurons. In addition, LEPRb activation stimulates the melanocyte-stimulating hormone (α-MSH) via POMC, further reducing food intake. Leptin also interacts directly with kisspeptin neurons [43,44]. Based on the energy status and circulating leptin levels, those processes modulate GnRH neurons and can disrupt the pulsatile character of GnRH release, which directly affects the frequency and amplitude of gonadotropin pulses [19,37,45,46]. The cells of the pituitary gland also respond to changing leptin concentrations, showing LEPR expression. Existing evidence demonstrates the stimulatory effect of leptin on activin mRNA in gonadotropes, which is crucial for follicle-stimulating hormone (FSH) synthesis [47,48]. At the initial stage of the menstrual cycle, FSH determines the development of ovarian follicles and stimulates them to secrete estradiol, providing positive feedback to the CNS and enhancing the downstream signaling from GnRH neurons and gonadotropes [48]. It has been observed that the serum leptin level changes with the menstrual cycle phases, reaching its peak just before ovulation and coinciding with the luteinizing hormone (LH) surge [49,50,51,52]. Studies in non-primate animals and in vitro research showed a dose-dependent effect of leptin on LH, FSH and prolactin release by the anterior pituitary, which was modulated by nitric oxide (NO) [52,53,54]. Based on the observation that the LH surge activates inflammatory pathways in ovarian follicles [55] and the pro-inflammatory character of leptin, Salem at al. suggested that the increased leptin concentration in the periovulatory period “might be related to inflammatory responses associated with ovulation” [56]. Possibly, leptin also plays an important regulatory role in the gonadotropes—it suppresses miRNA (miR-581/669d) and Musashi1 (MSI1) protein, which inhibit *Gnrhr* mRNA translation. Therefore, by exhibiting de-repressing function and promoting *Gnrhr* mRNA translation, leptin enables a proper response to GnRH in the anterior pituitary and determines the physiological FSH and LH fluctuations [48].

The occurrence of LEPR was also reported in oocytes as well as granulosa, theca and interstitial cells in animal models and human ovaries [57]. Leptin is present in the follicular fluid and its concentrations align with the serum concentrations, reaching higher levels in women with obesity [20]. By suppressing estradiol production and inhibiting dominant follicle development and oocyte maturation, elevated leptin levels may exert an inhibitory effect on ovaries and lead to ovulatory disorders [29]. A study by Hong at al. from 2022 revealed a significant correlation between the leptin levels and the anti-Mullerian hormone (AMH) levels in in vitro (IVF) patients undergoing ovarian stimulation using the GnRH antagonist protocol. The leptin concentrations were measured in preovulatory follicles during oocyte retrieval in women with different baseline AMH levels and were remarkably higher in women with normal AMH concentrations compared to low or high levels. Based on these results, the authors suggested that leptin could serve as a potential marker of oocyte maturity and reproductive potential [58]. Additionally, the meta-analysis by Al-Aqbi at al. from 2020 summarized the available data on the association of leptin concentrations in both follicular fluid and serum and their association with IVF outcomes expressed as the occurrence of pregnancy. At the time of oocyte collection, the follicular fluid leptin levels were significantly lower in the pregnant women in comparison to the non-pregnant group. However, no statistical difference in the serum leptin levels between the groups was reported. The treatment used in the included studies was a daily dose of FSH or recombinant FSH (rFSH) until the follicles reached about 17–18 mm and 10,000 IU of human chorionic gonadotropin (hCG) after they reached the expected diameter. This meta-analysis indicated that leptin could be also considered a predictor of pregnancy outcome following IVF [59].

It should be noted that both states, leptin excess as well as leptin deficiency, contribute to reproductive disfunction. The hyperleptinemia observed in common obesity, polycystic ovary syndrome (PCOS) and type 2 diabetes mellitus (T2DM) can be associated with central leptin resistance and have a direct impact on the gonads. On the other hand, lack of leptin activity, signaling insufficient energy resources, can also impair fertility, as observed in congenital leptin deficiency, lipodystrophy, hypothalamic amenorrhea and type 1 diabetes [60].

### 2.2. Adiponectin

Adiponectin (APN) is the most abundant circulating adipokine secreted by WAT [20,28,30]. Its concentrations are inversely correlated with a degree of obesity and the amount of adipose tissue [61,62]. APN modulates glucose uptake and has a protective role against insulin resistance (IR), which is one of the long-term complications of obesity and can lead to T2DM development.

APN consists of 244 amino acids and has a molecular weight of 28 kDa in humans (247 amino acids and 20 kDa in mice) [63]. Encoded by the *ADIPOQ* gene and classified into the collagen superfamily, its function resembles complement factor C1q and TNF [64]. Adiponectin exists in three oligomeric complexes: a low-molecular-weight trimer (APN-LMW), a medium-molecular-weight hexamer (APN-MMW) and a high-molecular-weight multiform (APN-HMW) [20,65]. Adiponectin acts through three different receptors, each binding a different molecular form: APN-LMW binds to AdipoR1, APN-MMW binds to AdipoR2 and APN-HMW binds to T-cadherin, which was discovered most recently [28]. APN triggers various signaling cascades, which involve AMPK and p38 MAPK phosphorylation, peroxisome-proliferator-activated receptor (PPAR) α and γ, PPAR γ-coactivator-1α (PGC-1α) and IRS-1 [22,66]. Moreover, adiponectin regulates nitric oxide synthase (eNOS) production in endothelial cells, which mediates angiogenesis and serves as a vascular protective agent [67,68]. The main target sites for APN action are the skeletal muscles and the liver, where it enhances glucose uptake, at the same time diminishing hepatic gluconeogenesis and stimulating fatty acid β-oxidation in myocytes via AMPK-mediated pathway. In muscles, APN is responsible for glucose transporter type 4 (GLUT4) translocation and stimulates lactate production. It promotes myocytes to shift toward catabolic pathways and adenosine triphosphate (ATP) production [22]. Therefore, by improving the insulin sensitivity profile and reducing triglyceride aggregation, APN exerts its anti-diabetic and anti-atherosclerotic properties [12,20,67]. Although adiponectin is mainly considered an anti-inflammatory adipokine, it can also operate as a pro-inflammatory factor. These opposite effects may depend either on the APN concentrations, on which of different kinds of extracellular receptors on the immune cells would be currently stimulated or on the proportion of coexisting molecular forms [28,69]. When showing its anti-inflammatory aspect, it limits ubiquitous chronic inflammation by promoting macrophage polarization toward the M2 phenotype and suppressing TLR4 expression [70]. On the contrary, revealing its pro-inflammatory character, it can lead to NF-κB activation and increased cytokine production by myocytic cells and macrophages, as well as initiate their conversion into the M1 type [64,71].

The major function of adiponectin is sensitizing tissues to insulin and diminishing hepatic glucose production. Obesity, in which the adiponectin levels are decreased, leads to growing IR and hyperinsulinemia, which stimulates ovarian androgen production, disrupting hormonal balance. It is one of the mechanisms underlying the development of PCOS, which is very often associated with increased body weight. Furthermore, IR can directly affect HPG axis. By altering the GnRH, FSH and LH pulses, it results in menstrual disorders and may cause anovulation [19]. A more detailed impact of hyperinsulinemia and androgen excess on reproduction will be discussed later in this article.

Despite primarily targeting the skeletal muscles and the liver, adiponectin has also been detected in the cardiomyocytes, bones, adrenal glands, lymphoblasts, placenta and gonads [63]. Although receptors for adiponectin have been found in all the peripheral reproductive tissues, its direct role associated with binding to the receptors remains unclear. Some studies suggest that APN might modulate steroidogenesis in granulosa and theca cells and affect gametogenesis [30,66]. In addition, both AdipoR1 and AdipoR2 expression has been reported in the hypothalamus and pituitary gland, pointing toward its potential direct impact on the HPG axis. After many years of controversies, it has been proved that APN can cross the BBB [72,73]. Leptin and adiponectin might show a synergistic effect on metabolic regulation—LEPRs and AdipoRs share overlapping signaling pathways, such as JAK2/STAT3, IRS1/2, AMPK and PI3K [74]. The role of APN in the central regulation of energy management and reproduction is complex. APN regulation of anorexigenic POMC activity depends on the glucose levels—in hypoglycemia, it has a stimulatory effect through PI3K signaling and an inhibitory effect through AMPK signaling at high glucose levels. On the contrary, APN inhibits orexigenic NPY/AgRP neurons independently of the circulating glucose concentrations [75]. Moreover, studies by Wen at al. demonstrated the suppressive impact of APN on GnRH secretion and kisspeptin gene transcription in the hypothalamus, which can alter gonadotropin release from the pituitary gland and secondarily impair ovarian function in women [76,77].

### 2.3. Resistin

Resistin is another important adipokine, involved in metabolic overturn and regulating energy homeostasis. In humans, this 12.5 kDa cysteine-rich polypeptide, encoded by the RETN gene, consists of 108 amino acids and forms high- and low-molecular-weight complexes [12,66,78]. Its expression has been detected in the AT; however, unlike leptin and adiponectin, resistin is highly expressed in bone marrow macrophages and peripheral blood mononuclear cells (PBMCs) [79,80]. This pro-inflammatory adipokine has been linked to obesity, IR and PCOS, showing a positive correlation with the BMI and the amount of VAT [81]. Resistin acts directly on adipocytes, inhibiting their differentiation and insulin-induced glucose uptake [12,20]. By inducing a suppressor of cytokine signalling-3 (SOCS-3), resistin facilitates the development of IR [22,82]. SOCS-3 is also involved in promoting inflammation and suppressing the JAK2/STAT3 pathway, which lead to central leptin resistance [83]. Moreover, by promoting vascular cell adhesion molecule 1 (VCAM-1) and MCP-1 expression in endothelial cells, resistin enhances foam cell formation and vascular smooth muscle cell apoptosis, which substantially contribute to the development of atherosclerosis—one of the primary complications of obesity [65]. The pleiotropic function of resistin includes modulating hepatic function by AMPK activation, regulating cholesterol metabolism via the PPARγ-dependent PI3K/Akt signaling pathway in macrophages and enhancing their proinflammatory effect by triggering NF-κB via JNK and p38 MAPK [12]. Receptors for resistin still have not been identified; however, potential molecular targets for resistin involve decorin (DCN), mouse receptor tyrosine kinase-like orphan receptor 1 (ROR1), TLR4 or adenylyl cyclase-associated protein 1 (CAP1) [66].

Although the expression of resistin has been discovered in the hypothalamus and pituitary gland, its role in regulating gonadotropin secretion has not been fully elucidated yet [84]. In the hypothalamus, resistin activates the TLR4 receptor, initiating a downstream signaling cascade. Maillard at al. confirmed the presence of resistin mRNA and its expression in all the cell types of the murine anterior pituitary, especially gonadotrophs. However, it has been detected in neither the intermediate nor posterior lobe. The results of this study demonstrated that resistin directly affects the pituitary gland via the AMPK and ERK1/2 signaling pathways, decreasing basal LH secretion in a dose-dependent manner, which points toward the potential role of resistin in fertility impairment [85]. Resistin is also present in the gonads, which has been reported in many different species, including humans. Expressed in granulosa cells, theca cells and oocytes, its potential role involves follicular development and proliferation [66]. Resistin shows pleiotropic activity and affects a broad range of cells in an autocrine, paracrine, and endocrine manner [86]. In ovaries, it promotes steroidogenesis by activating an array of steroidogenic enzymes such as 3β-hydroxysteroid dehydrogenase (HSD3B), 17β-hydroxysteroid dehydrogenase (17βHSD), CYP17A1 and CYP11A1 (cytochrome P450 enzymes). Resistin increases progesterone, androstenedione and testosterone secretion but does not affect estrogen and CYP19A1 expression in ovarian follicles [87]. On the other hand, in primary human granulosa cells, resistin inhibits both progesterone and estrogen secretion induced by insulin-like growth factor 1 (IGF-1), lowering the P450scc and P450 aromatase levels. At the same time, phosphorylation of MAPK ERK1/2 is reduced and there is no change in the steroidogenic acute regulatory protein (STAR) and HSD3B protein concentrations [88].

### 2.4. Visfatin

Visfatin, or nicotinamide phosphoribosyltransferase (NAMPT), is a 52 kDa cytokine, which also acts as an enzyme. In the past, it was referred to as pre-B-cell colony-enhancing factor (PBEF) due to its impact on B-cell precursors. Although it is predominantly secreted in VAT by macrophages in response to inflammation, it can also be expressed in the lymphocytes, bone marrow, hepatocytes, pancreas and muscle cells, trophoblast and fetal membranes [12,20,89]. Visfatin is present in two forms, which operate through different mechanisms. Acting as an enzyme, its intracellular form is involved in nicotinamide adenine dinucleotide (NAD+) biosynthesis and enhancing sirtuin 1 (SIRT1) deacetylase activity in mitochondria [22,90]. The NAMPT/NAD+/SIRT1 pathway is responsible for adaptive responses and promoting lipid storage as a response to fasting [91]. However, under excessive calorie intake, these processes may be impaired, leading to T2DM development and obesity progression [90,92]. The extracellular isoform acts like a classical hormone and cytokine, showing endocrine, paracrine, and autocrine action [93,94]. Visfatin stimulates glucose uptake in adipocytes and myocytes, inhibits hepatic glucose release, participates in lipogenesis and, by regulating JAK2/STAT3 and IκB kinase/NF-κB (IKK/NF-kB) signaling, decreases insulin sensitivity [94,95]. However, the insulin-mimicking effect of visfatin and its precise role in metabolic regulation still remain controversial. Contrary to resistin, it promotes adipocyte differentiation [96,97]. Visfatin certainly played an important role throughout evolution as it is highly conserved between various species [85,98]. The visfatin-specific receptors still have not been identified, but some authors suggest that it might show affinity for insulin receptors [78,99]. Visfatin is also considered a pro-inflammatory cytokine, which shows affinity for the TLR-4 receptor and leads to increased release of inflammatory cytokines, such as TNF-α, IL-6, and IL-1β, which further stimulate its expression, creating a feedback loop [22,65,100]. Moreover, visfatin impairs endothelial function via activating the NF-kB pathway and supports macrophage adhesion to endothelial cells by enhancing E-selectin, intercellular adhesion molecule 1 ICAM-1 and VCAM-1 expression [81,101]. Through promoting cholesterol accumulation in macrophages and accelerating atherosclerosis, it is involved in the pathogenesis of cardiovascular events, including myocardial failure and ischemic stroke [65,98,102,103].

Elevated levels of circulating serum visfatin are closely related to conditions such as obesity, IR and T2DM [104,105]. Furthermore, high visfatin concentrations seem to be intrinsic to the pathogenesis of PCOS, independently of the degree of concomitant obesity [106,107]. Despite the connection between visfatin and insulin management disruption, followed by an altered androgen to estrogen ratio, visfatin also regulates reproductive function by acting at all the levels of the HPG axis [85,108,109]. This adipokine has been detected in the areas of the hypothalamus responsible for GnRH production. Its expression increases in pregnancy and seems to change with each phase of the menstrual cycle, reaching the highest rate in the early-luteal phase [108,110]. In a study by Szymanska at al., visfatin was observed in the porcine pituitary gland in both the anterior and posterior lobes, varying in terms of the gene and protein expression patterns. Using immunofluorescence, the localization of visfatin was confirmed in gonadotrophs, corticotrophs, thyrotrophs, lactotrophs and somatotrophs [108]. In mice, it was only detected in the anterior and intermediate lobes. Mostly present in gonadotroph cells, it suppresses LH release through the AMPK pathway [85]. This adipokine has also been found in cerebrospinal fluid (CSF) [111]. Although visfatin can cross the BBB, its concentration in CSF is equal to approximately just 10% of the plasma level. It has been suggested that the local production of visfatin has a major influence on central nervous system regulation and visfatin secreted by peripheral tissues plays a secondary role [110]. In addition, visfatin is also expressed in the ovaries and can be found in oocytes as well as granulosa and theca cells [112]. Visfatin takes part in follicular development and shows greater expression in their growth phase than in mature ones [89].Its beneficial impact on oocyte quality and fertility may partially result from promoting ovarian angiogenesis [113]. Additionally, in opposition to resistin, by increasing STAR and HSD3B expression as well as MAPK3/1 phosphorylation, visfatin promotes estradiol and progesterone secretion in primary human granulosa cells, which strengthens the IGF-1–induced effect [114].

### 2.5. Omentin

Omentin is an anti-inflammatory adipokine that increases insulin sensitivity, regulates lipid metabolism and acts as an antioxidant [20,81]. It exists in two homologous forms, omentin-1 and omentin-2, which are encoded by adjacent genes in the area of chromosome 1 linked to T2DM [115]. Omentin-1, also known as intelectin-1, endothelial lectin HL-1 or intestinal lactoferrin receptor, consists of 313 amino acids and has a molecular weight of 35 kDa [12,116]. It is a major circulating form, mainly secreted by SVF cells in omental VAT. Although preferentially expressed in VAT, it is also present in SAT at lower concentrations and partially underlies the differences in the physiology of those two tissues [20]. Obesity, IR, T2DM and some inflammatory diseases, such as psoriasis or inflammatory bowel disease, have been linked to significantly lower omentin-1 serum levels [117,118,119]. One of the main functions of omentin is promoting insulin-stimulated glucose uptake in adipocytes through suppression of the mTOR signaling pathway and IRS activation [120,121]. Moreover, omentin-1 increases Akt and AMPK phosphorylation in macrophages, reducing their infiltration and foam cell formation [115,122]. It also stabilizes plaques by binding to the αvβ3 and αvβ5 integrin receptors in macrophages [123] and improves endothelial function through increasing NO production [124]. Despite directly suppressing the function of macrophages, omentin-1 exerts its anti-inflammatory function by inhibiting TNF-α-mediated cyclooxygenase 2 (COX-2) expression, activating JNK via AMPK/eNOS/NO and potentially blocking the ERK/NF-κB pathway [125,126]. The protective impact of omentin-1 on the cardiovascular system limits atherosclerosis and makes it a promising candidate for potential use in the treatment of vascular complications associated with obesity [124].

The empirical data concerning the impact of omentin on higher levels of the HPG axis are very limited. In a study from 2013, Brunetti at al. described the orexigenic role of omentin-1 manifested through suppressing the cocaine- and amphetamine-regulated transcript (CART) and corticotropin-releasing hormone (CRH) gene expression and stimulating norepinephrine synthesis in the hypothalamus. Despite the hypothesis that peripheral mechanisms might be involved in the process, it was proved that omentin-1 can act directly on hypothalamic neurons [127]. However, the effect of omentin-1 on GnRH release remains unknown. Recently, Respekta at al. demonstrated omentin-1 expression in the porcine anterior pituitary gland, which depended on the LH, FSH and GnRH concentrations and might suggest the potential influence of the menstrual cycle phases on omentin-1 secretion in humans [128]. The role of omentin-1 in the central regulation of reproductive function is still unclear and more research is needed to assess its exact role and mechanism of action. In addition, omentin-1 is present in the placenta and ovaries, where its mRNA shows high expression [129,130]. According to Dupont et al., omentin-1 takes part in folliculogenesis. By interacting with visfatin, it stimulates IGF-1R signaling and steroidogenesis initiated by IGF-1 [116]. A series of experiments from 2024 conducted by Sirotkin at. proved that omentin can regulate basic ovarian cell functions. Enhancing the proliferation:apoptosis rate in granulosa cells, it increases cell proliferation and viability. Furthermore, it was demonstrated that omentin limited progesterone secretion by both granulosa cells and ovarian fragments. On the contrary, in the case of estradiol, omentin reduced its release by granulosa cells but stimulated it in ovarian fragments [131]. In luteinized granulosa cells, the omentin concentrations are responsive to FSH, IGF-1 and insulin regulation, which points to its direct connection to the menstrual cycle and carbohydrate metabolism [116].

Independently of the BMI, omentin-1 expression is lower in women with PCOS than in those without PCOS [132]. Moreover, among those diagnosed with PCOS, the omentin-1 concentrations are lower in the early follicular phase when menstrual cycles are irregular compared to regular menstruation. The omentin-1 levels also show an inverse correlation with the Homeostatic Model Assessment for Insulin Resistance (HOMA-IR) and fasting insulin [133]. They increase after metformin and liraglutide treatment, which are used in IR and T2DM [134,135,136]. Omentin-1 expression is modified in inflammatory states, such as obesity, which leads to low-grade systemic inflammation. Therefore, this adipokine might be considered a factor predictive of metabolic consequences and comorbidities of obesity, including metabolic syndrome and PCOS [133,137]. A meta-analysis by Tang at al. from 2017 summarizes numerous studies, indicating the close association of omentin-1 with insulin sensitivity and its potential link to the pathogenesis of PCOS [132], which may result in pregnancy complications and infertility issues [138].

In conclusion, the most studied effect of adipokines on the reproductive system is the impact of leptin and adiponectin on the hypothalamus, alerting GnRh, FSH and LH pulsatory secretion. However, most of the adipokines or their receptors are also expressed in the gonads, altering follicular development and hormone secretion directly. Moreover, several adipokines, such as adiponectin, resistin or visfatin, exert a profound impact on inflammatory processes and insulin resistance development correlated with anovulatory disorders such as polycystic ovary syndrome.

## 3. Role of Kisspeptin in HPG Axis Regulation

Chronic obesity leads to suppression of kisspeptin neurons, which link food intake and reproduction, managing the energy balance. Kisspeptin is an important mediator providing fundamental crosstalk between both circuitries and plays a key role in regulating hypothalamic function. Excessive adipose tissue, responsible for an altered metabolic state, leads to inhibited kisspeptin secretion, lower LH pulses and, consequently, anovulatory conditions [139]. However, in women with PCOS, the kisspeptin concentrations are higher than in the healthy control group, independently of obesity status. Moreover, in non-obese PCOS patients, the levels of kisspeptin are more elevated than in obese PCOS women. In comparison to the controls, the kisspeptin levels are positively correlated with LH, AMH and testosterone in the total PCOS group, indicating the possible regulatory role of kisspeptin in PCOS [140].

Kisspeptin, also named metastatin, is a product of the KISS1 gene, which was first described as a human metastasis-suppressor gene in malignant melanoma cells that had lost the ability to metastasize [141,142].

Kisspeptin neurons are mainly located in the anteroventral periventricular/periventricular preoptic nucleus (AVPV/PeN) and ARC. They are stimulated by α-MSH produced by POMC neurons and can also be influenced by estrogens and leptin. The pulsatile secretion of kisspeptin regulates the pulsatile secretion of GnRH and LH [143,144]. Therefore, the hormonal activity of adipose tissue, through leptin, exerts a permissive effect on the production of GnRH in the hypothalamus and regulates maturation and reproduction [144]. Neurons localized in the AVPV/PeN participate in conveying the positive and negative effects of estrogens on GnRH secretion [145,146,147,148,149]. LH and FSH modulate the production of steroid hormones in the gonads, which in turn provide feedback regulation to the brain via kisspeptin [17]. Novaira at al. showed that kisspeptin causes specific histone modifications in murine GnRH neuronal cell lines (GT1-7 cells), leading to the increased expression of the GnRH gene and subsequent release of this hormone [150]. Moreover, studies on kisspeptin-54 and the kisspeptin receptor agonist MVT-602 demonstrated that they cause a surge of LH with similar parameters to the pre-ovulatory LH surge, which may indicate their utility in inducing ovarian stimulation during female infertility treatment [151,152]. A recent study by Villa at al., published in May 2024, confirmed the lower pulse frequency of LH secretion in obesity and demonstrated the downregulation of α-MSH receptors, regulating kisspeptin neurons. At the same time, pituitary responsiveness to GnRH remained unaffected [139].

Kisspeptin not only affects the HPG axis directly but also acts peripherally on female reproductive organs. The expression of genes encoding both kisspeptin and its receptor GPR54 was proved in the ovaries, epithelium cells in the oviduct and endometrium [153]. Administration of kisspeptin-54 elevates expression of genes encoding the FSH receptor, LH receptor, CYP19A1 (aromatase), STAR, estrogen receptor 1 and 2 (ESR1 and ESR2) and Inhibin A in granulosa lutein cells, which participate in the production of steroid hormones in ovaries and regulate the menstrual cycle [154].

Obesity causes a chronic inflammatory state in the human body. Via leptin resistance and promoting inflammatory response, this morbid condition downregulates kisspeptin expression and shows a suppressive effect on the HPG axis [155]. Iwasa at al. reported that systemic administration of a high dose of pro-inflammatory lipopolysaccharide (LPS) reduced the expression of the KISS1 gene and the serum level of LH in rats [156]. Another study, using primary human fetal hypothalamic cells, demonstrated that GnRH-releasing neurons inhibit the expression of the KISS1 receptor under the influence of TNF-α, which is produced, among others, in AT. However, due to the fetal origin of the examined cells, this model may not accurately reflect the GnRH physiology in adults [157]. Kisspeptin secreted by adipocytes acts as a paracrine factor affecting the hormonal activity of AT. It has been reported that administration of exogenous kisspeptin-10 increases the concentration of adiponectin in plasma but does not change the levels of leptin and resistin in rhesus monkeys [158].

Kisspeptin remains the main regulatory factor of GnRh secretion in the hypothalamus, promoting puberty development and sustaining effective ovary function during reproductive years. Appropriate adipose tissue function, expressed by leptin levels and sensitivity, enables kisspeptin to fulfill its role as one of the key factors regulating the reproductive system.

## 4. Neuroinflammation

For years, the brain was regarded as an immunologically privileged organ, protected by the BBB and with limited access to the peripheral immune system. However, recent advances in diagnostic methods and imaging provided new insight into the role of the CNS as an immunologically active site, which synchronizes both immune privilege and surveillance [159].

Diet-induced metabolic stress, observed in obesity, changes neuronal activity and has a particular impact on microglia, which are resident immune cells of the CNS involved in modulating brain circuitry, synaptic plasticity, regeneration and neurogenesis [17,160,161]. Microglia can be activated not only by injuries, infection or disease but also through stimulation from LPS, FFA, carbohydrates and leptin [160,162]. Those neuroinflammatory changes in glial functions were detected specifically in the ARC and median eminence (ME) in the hypothalamus, but not in the ventromedial hypothalamus (VMH) or other brain structures [162]. Consequently, the altered morphology of microglial cells and their accumulation, called “microgliosis”, lead to hypothalamic dysregulation and disrupted microglial–neuronal crosstalk, which is fundamental for maintaining metabolic homeostasis and thus reproduction [163]. Microglia express the CX3C motif chemokine receptor 1 (CX3CR1) and respond to neuronal signaling through the fractalkine (CX3CL1) pathway in a healthy brain [164]. Dorfman et al. [165] observed that a high-fat diet (HFD) leads to a reduction in CX3CR1 and CX3CL1 expression in mice, which translates to increased microglia activation and local inflammation. This effect was strongly expressed in males, while females were more resistant. Female Cx3cr1gfp/gfp knock-in mice, in which the Cx3cr1 gene has been knocked out and replaced by Gfp, showed a “male-like” response to HFD and increased susceptibility to diet-induced obesity. To prove that the difference in response to HFD resulted from a genetic change, and not from abnormalities in the function of the female gonads and their hormonal activity, the researchers evaluated the normality of the ovarian structure, the estrous cycle and the estradiol serum levels. This pointed to sex differences in response to HFD, independent of ovarian estrogen and possibly resulting from sex-specific alternations in intracellular communication in the CNS [165]. Lainez at al. indicated that it might also arise from larger visceral fat accumulation in males and thus increased inflammation [17]. Moreover, in a state of nutrient excess, microglial cells are triggered by TLR4, leading to activation of the JNK, NF-kB and COX-1 pathways, which initiates immune response and secretion of pro-inflammatory cytokines such as TNF-α, IL-1 and IL-6 [18]. An increased amount of VAT, which is a reservoir of macrophages, corresponds with higher infiltration rate of macrophages into the hypothalamus in response to resident microglia cytokine secretion and signaling, specifically in males. These macrophages express the C–C chemokine receptor type 2 (CCR2) and share identical pro-inflammatory and metabolic markers with AT macrophages [166,167]. Astrocytes are other cells participating in sustaining neuroinflammation and activating microglia, which, in turn, stimulate them back. Additionally, vascular endothelial growth factor (VEFG), produced by astrocytes, further disrupts the BBB and thus facilitates FFA flow into the hypothalamus [18]. Another mechanism modulating neuroinflammation is associated with vagus nerve cholinergic signaling, also involved in the regulation of cognition. The vagus nerve is a major link connecting the brain with peripheral organs such as the liver, gastrointestinal track and the pancreas. Therefore, it plays an important role in maintaining metabolic homeostasis and controlling inflammation via the vagus nerve-based inflammatory reflex [168].

The hypothalamus is surrounded by areas containing fenestrated capillaries, which, together with obesity-related alterations in the BBB, facilitate cytokine and macrophage infiltration. POMC, NPY and kisspeptin neurons are located in the ARC, close to the ME and organum vasculosum laminae terminalis (OVLT), which makes them easily accessible by circulating pro-inflammatory cytokines [167]. Moreover, the proximity of GnRH neurons enhances their susceptibility to metabolic changes and impaired signaling. Inflammatory mediators associated with increased fat mass may also impact the HPG axis directly on a pituitary level, altering the gene expression of gonadotropin units. It was documented that both acute and chronic inflammation leads to the downregulation of GnRH and LH secretion via the TLR2/4 pathway, while in the case of FSH, only a prolonged inflammation state inhibits its production [169,170]. Other mechanisms involved in reproductive function impairment initiated by neuroinflammation are synaptic remodeling, changes in GnRH network connectivity and altered expression of synaptic receptors and neuropeptides [17].

There are sex-specific differences in the hypothalamic response to immunological and metabolic changes observed in obesity. Females seem to be less susceptible to the effect of the endocrine function of AT. This phenomenon may be attributed to various factors, such as the WAT distribution [167], epigenetics and gene expression, as well as differences at the level of intracellular signaling and microglia activation [165]. The protective function of ovarian estrogen was proved in the case of diet-induced obesity, but there is no evidence indicating its role in hormonal and immunological alterations [167]. In women, the excess of androgens contributes to metabolic disfunction, promoting IR and T2DM development, which also exacerbate oxidative stress and hypothalamic inflammation. Androgen concentrations within the normal range are crucial to ensuring proper *pomc* expression and leptin-mediated BAT thermogenesis, and thus, to maintaining energy homeostasis [171].

## 5. Hyperinsulinemia and Sex Hormones

The primary causes of obesity include hyperinsulinemia and IR, in addition to hyperandrogenism and changes in steroidogenesis in individuals with ovarian stromal hyperplasia. Dysfunctional obese adipose tissues release excessive amounts of FFA, ROS and pro-inflammatory cytokines, leading to early IR. Increased FFA and dietary lipids enter non-adipose cells, producing toxic lipids like ceramides, which disrupt mitochondria, the endoplasmic reticulum (ER), and lysosomes. Chronic obesity and overnutrition cause the malfunction of these organelles, leading to cellular function impairment, systemic dysfunction and apoptosis. Disrupted insulin sensitivity and glucose homeostasis, which increase systemic FFA and lipid deposition in non-adipose organs, cause further pro-inflammation and ROS production. Immune cells accumulate in inflamed areas to resolve complications but can also contribute to inflammation. ER stress and lysosomal dysfunction, mediated by Ca^2+^ and ROS, trigger inflammation, which is crucial for the onset of IR [13]. Both hyperinsulinemia and hyperandrogenism impact ovarian function in both obese and non-obese women. The mechanism of IR in women with PCOS may be linked to the excessive serine phosphorylation of the insulin receptor. In particular, the serine phosphorylation of IRS1 and IRS2 inhibits insulin signaling [172]. Additionally, hyperinsulinemia is one of the factors lowering the hepatic production of SHBG, resulting in elevated levels of circulating androgens [173]. Insulin also exerts a specific influence on steroidogenesis through its interaction with insulin receptors, thereby stimulating theca cells to produce androgens and promoting the proliferation of stromal cells [20].

Another proposed mechanism by which hyperinsulinemia induces hyperandrogenism in PCOS patients, both obese and non-obese, involves IGF-1. This growth factor is generated by human ovarian tissue and possesses receptors within the ovaries [20,174].

Insulin amplifies LH activity in granulosa cells by stimulating steroidogenesis and inhibiting mitosis in the preovulatory follicle [175,176]. This dual effect of insulin leads to an unfavorable environment that impedes follicle growth, resulting in premature luteinization and follicular arrest. These conditions can result in menstrual cycle irregularities and oligo-anovulation, which is particularly associated with obesity [177]. Additionally, increased estrogen production from peripheral conversion disrupts the HPG axis, making excess estrogen and hyperandrogenism significant contributors to anovulation in these patients.

Excessive accumulation of visceral adipose tissue is associated with hyperinsulinism and insulin resistance. High insulin levels increase ovarian androgen production directly or by IGF-1 receptors. Moreover, obesity decreases SHBG production, additionally enhancing the free androgen availability. Androgens, in turn, favor visceral adipose tissue accumulation and further development of IR in obese and PCOS patients.

### Polycystic Ovary Syndrome

Polycystic ovary syndrome is a multifactorial endocrine disorder characterized by reproductive, metabolic, and psychological symptoms, which affects about 8–13% of women of reproductive age [178]. PCOS should be diagnosed according to the updated evidence-based Rotterdam criteria. For adults, diagnosis requires the presence of at least two of the following: (1) clinical/biochemical hyperandrogenism, (2) ovulatory dysfunction, or (3) polycystic ovaries observed on ultrasound or elevated AMH levels, after ruling out other potential causes. In cases where irregular menstrual cycles and hyperandrogenism are evident, ultrasound or AMH testing is not necessary for diagnosis. In adolescents, both hyperandrogenism and ovulatory dysfunction must be present for diagnosis, as ultrasound and AMH testing lack specificity and are therefore not recommended [179]. In about 70% of cases, PCOS remains undiagnosed, which delays the implementation of proper treatment aimed at reducing the metabolic risk and regulating ovarian function [180].

Various studies investigated the epidemiological correlation between obesity and PCOS, indicating that a significant proportion of women with PCOS are also classified as overweight or obese [181,182,183,184,185,186]. PCOS stands as a prevalent cause of menstrual disorders, anovulation and unfavorable outcomes of ART among women of reproductive age, stemming from the prevalent hormonal imbalance and altered ovarian morphology.

Research in animal models provided significant insights into the pathophysiology of PCOS, particularly in terms of understanding the role of hyperandrogenism. For example, rodent models treated with androgens such as dihydrotestosterone (DHT) have been extensively used to mimic the hyperandrogenic state observed in PCOS. Studies in these models demonstrate that chronic exposure to elevated androgen levels leads to reproductive and metabolic disturbances akin to those seen in human PCOS [187]. Caldwell et al. developed the first letrozole and long-term DHEA murine models and compared them with prenatal and long-term DHT-androgenized mice. Their research revealed that only the in vivo increase of the potent androgen DHT, as opposed to the proandrogen DHEA, results in characteristics in mice that mimic PCOS. These characteristics include irregular estrous cycles with oligoanovulation, polycystic ovaries, obesity, dyslipidemia, and hepatic steatosis [188]. Additionally, these animal models have shown that hyperandrogenism can induce changes in the hypothalamic–pituitary–ovarian (HPO) axis, contributing to the dysregulation of gonadotropin secretion. This highlights the critical role of hyperandrogenism in the etiology of PCOS. Furthermore, animal studies have explored the impact of hyperandrogenism on neuroinflammation. For instance, androgenized rodent models have shown increased brain inflammation markers, including elevated cytokine levels and microglial activation, suggesting that hyperandrogenism may contribute to the neuroinflammatory processes observed in PCOS. These findings align with clinical observations linking PCOS with an increased risk of mood disorders and cognitive impairments, potentially mediated by neuroinflammatory pathways [189,190].

Insulin resistance and a tendency toward visceral adipose tissue accumulation, as observed in the majority of PCOS patients, contributing to the development of the disease, were discussed previously. Resistin appears to also be involved in the pathogenesis of PCOS. Some studies suggested that women with PCOS may have a specific resistin gene polymorphism linked to the BMI, emphasizing the connection between obesity and PCOS [191,192]. A randomized placebo-controlled study by Majuri at al. further supported these findings by proving that treating overweight women with PCOS using the insulin sensitizer rosiglitazone significantly decreased their serum resistin levels [193]. Independently of obesity, Dumesic at al. found that women with normal weight and PCOS have increased adipose IR and altered gene expression in the subcutaneous abdominal adipose stem cells (ASCs), which is associated with hyperandrogenism [194].

Polycystic ovary syndrome’s etiopathogenesis is multifactorial. However, obesity and the role of adiponectin, resistin and visfatin in insulin resistance occurrence have been demonstrated. Insulin resistance, in turn, is perceived as one of the main metabolic factors favoring PCOS development.

## 6. Future Perspectives

There has been astonishing progress in our knowledge of the key facets of adipose tissue products and their effect on the female reproductive system. Apart from a growing understanding of the adipokines’ action on the hypothalamus–pituitary–gonadal axis, both central as well as peripheral, there is accumulating evidence of the role of endocrine disruptors, microorganisms, ambient temperature or sleep debt in the development of obesity [195]. Moreover, intrauterine and epigenetic factors can lead to permanent changes in the developing fetus. Maternal leptin and insulin levels may be key factors affecting the birth weight and future predisposition to obesity of the offspring [195]. The effect of maternal obesity in pregnancy on specific aspects of the offspring’s reproductive function is less clear [196].

While the anatomical distribution and several functional features of the main players connecting adipose tissue and the function of the reproductive system have been established in the last few decades, we still need studies on different adipokines, inflammatory factors, kisspeptin and insulin, and their receptors, to understand more correlates in this complicated landscape. Research may help to define the potential biomarkers of disease or novel targets of pharmacological intervention. The practical approach to utilize leptin as an anti-obesity agent failed in achieving its aim. The role of the insulin sensitizer metformin, has been long acknowledged in the treatment of T2DM and PCOS. There are several ongoing experimental and clinical tests on the medical application of kisspeptin [197]. Glucagon-like peptide 1 (GLP-1)-based therapies proved to be effective in the treatment of T2DM and obesity. In the context of the female reproductive system, it has been postulated that GLP-1 not only reverses some of obesity-induced influences but also seems to have a direct effect on the HPG axis [198]. Moreover, GLP-1 and its mimetics may exert anti-inflammatory and anti-fibrotic effects in the gonads and the endometrium [198].

## 7. Conclusions

The increasing prevalence of overweight and obesity is one of the emerging problems worldwide, posing a challenge to the healthcare system and consuming a great amount of resources needed to manage concurrent complications. The accumulation of excessive fat tissue and its endocrine function disrupts metabolic homeostasis, leading to reproduction impairment. Molecular mediators of obesity-related chronic low-grade systemic inflammation directly affect the HPG axis function at all levels by altering the intracellular communication and gene expression. Together with increasing IR and hyperandrogenism, inflammatory signaling contributes to hormonal imbalance, ovarian dysfunction, impaired oocyte maturation and differentiation, menstrual cycle irregularities and ovulatory disorders, resulting in pregnancy complications, difficulty conceiving, miscarriages and IVF failures. To address these problems, it is crucial to take into consideration the multifactorial etiology of obesity, greatly surpassing solely excessive calorie intake. Besides lifestyle changes, surgical treatment and pharmacology, more targeted interventions at the level of molecular pathways might be a promising solution to improve the reproductive outcomes in women with obesity.

## Data Availability

Not applicable.

## References

[1] WHO European Regional (2022). Obesity Report 2022.

[2] The Lancet Gastroenterol Hepatol (2021). Obesity: Another ongoing pandemic. Lancet Gastroenterol. Hepatol..

[3] Reyes-Farias M., Fos-Domenech J., Serra D., Herrero L., Sánchez-Infantes D. (2021). White adipose tissue dysfunction in obesity and aging. Biochem. Pharmacol..

[4] Zhu Q., An Y.A., Scherer P.E. (2022). Mitochondrial regulation and white adipose tissue homeostasis. Trends Cell Biol..

[5] Venkatesh S.S., Ferreira T., Benonisdottir S., Rahmioglu N., Becker C.M., Granne I., Zondervan K.T., Holmes M.V., Lindgren C.M., Wittemans L.B.L. (2022). Obesity and risk of female reproductive conditions: A Mendelian randomisation study. PLoS Med..

[6] Min Y.I., Gao Y., Anugu P., Anugu A., Correa A. (2021). Obesity and overall mortality: Findings from the Jackson Heart Study. BMC Public Health.

[7] Andreasson A., Carlsson A.C., Önnerhag K., Hagström H. (2017). Waist/Hip Ratio Better Predicts Development of Severe Liver Disease within 20 Years Than Body Mass Index: A Population-based Cohort Study. Clin. Gastroenterol. Hepatol..

[8] Khan I., Chong M., Le A., Mohammadi-Shemirani P., Morton R., Brinza C., Kiflen M., Narula S., Akhabir L., Mao S. (2023). Surrogate Adiposity Markers and Mortality. JAMA Netw. Open.

[9] Sun Y., Chen S., Zhang X., Pei M. (2019). Significance of Cellular Cross-Talk in Stromal Vascular Fraction of Adipose Tissue in Neovascularization. Arterioscler. Thromb. Vasc. Biol..

[10] Huh J.Y., Park Y.J., Ham M., Kim J.B. (2014). Crosstalk between adipocytes and immune cells in adipose tissue inflammation and metabolic dysregulation in obesity. Mol. Cells.

[11] Ren Y., Zhao H., Yin C., Lan X., Wu L., Du X., Griffiths H.R., Gao D. (2022). Adipokines, Hepatokines and Myokines: Focus on Their Role and Molecular Mechanisms in Adipose Tissue Inflammation. Front. Endocrinol..

[12] Kirichenko T.V., Markina Y.V., Bogatyreva A.I., Tolstik T.V., Varaeva Y.R., Starodubova A.V. (2022). The Role of Adipokines in Inflammatory Mechanisms of Obesity. Int. J. Mol. Sci..

[13] Ahmed B., Sultana R., Greene M.W. (2021). Adipose tissue and insulin resistance in obese. Biomed. Pharmacother..

[14] Saltiel A.R., Olefsky J.M. (2017). Inflammatory mechanisms linking obesity and metabolic disease. J. Clin. Investig..

[15] Zatterale F., Longo M., Naderi J., Raciti G.A., Desiderio A., Miele C., Beguinot F. (2019). Chronic Adipose Tissue Inflammation Linking Obesity to Insulin Resistance and Type 2 Diabetes. Front. Physiol..

[16] Yang S., Zhao M., Jia S. (2023). Macrophage: Key player in the pathogenesis of autoimmune diseases. Front. Immunol..

[17] Lainez N.M., Coss D. (2019). Obesity, Neuroinflammation, and Reproductive Function. Endocrinology.

[18] Ferreira-Hermosillo A., de Miguel Ibañez R., Pérez-Dionisio E.K., Villalobos-Mata K.A. (2023). Obesity as a Neuroendocrine Disorder. Arch. Med. Res..

[19] Zheng L., Yang L., Guo Z., Yao N., Zhang S., Pu P. (2023). Obesity and its impact on female reproductive health: Unraveling the connections. Front. Endocrinol..

[20] Silvestris E., de Pergola G., Rosania R., Loverro G. (2018). Obesity as disruptor of the female fertility. Reprod. Biol. Endocrinol..

[21] Coelho M., Oliveira T., Fernandes R. (2013). Biochemistry of adipose tissue: An endocrine organ. Arch. Med. Sci..

[22] Sahu B., Bal N.C. (2023). Adipokines from white adipose tissue in regulation of whole body energy homeostasis. Biochimie.

[23] Caër C., Rouault C., Le Roy T., Poitou C., Aron-Wisnewsky J., Torcivia A., Bichet J.C., Clément K., Guerre-Millo M., André S. (2017). Immune cell-derived cytokines contribute to obesity-related inflammation, fibrogenesis and metabolic deregulation in human adipose tissue. Sci. Rep..

[24] Nono Nankam P.A., Blüher M. (2021). Retinol-binding protein 4 in obesity and metabolic dysfunctions. Mol. Cell. Endocrinol..

[25] Helfer G., Wu Q.F. (2018). Chemerin: A multifaceted adipokine involved in metabolic disorders. J. Endocrinol..

[26] Guardado S., Ojeda-Juárez D., Kaul M., Nordgren T.M. (2021). Comprehensive review of lipocalin 2-mediated effects in lung inflammation. Am. J. Physiol. Lung Cell. Mol. Physiol..

[27] Obradovic M., Sudar-Milovanovic E., Soskic S., Essack M., Arya S., Stewart A.J., Gojobori T., Isenovic E.R. (2021). Leptin and Obesity: Role and Clinical Implication. Front. Endocrinol..

[28] Taylor E.B. (2021). The complex role of adipokines in obesity, inflammation, and autoimmunity. Clin. Sci..

[29] Pérez-Pérez A., Sánchez-Jiménez F., Maymó J., Dueñas J.L., Varone C., Sánchez-Margalet V. (2015). Role of leptin in female reproduction. Clin. Chem. Lab. Med..

[30] Kawwass J.F., Summer R., Kallen C.B. (2015). Direct effects of leptin and adiponectin on peripheral reproductive tissues: A critical review. Mol. Hum. Reprod..

[31] Liu J., Yang X., Yu S., Zheng R. (2018). The Leptin Signaling. Adv. Exp. Med. Biol..

[32] Gorska E., Popko K., Stelmaszczyk-Emmel A., Ciepiela O., Kucharska A., Wasik M. (2010). Leptin receptors. Eur. J. Med. Res..

[33] Evans M.C., Lord R.A., Anderson G.M. (2021). Multiple Leptin Signalling Pathways in the Control of Metabolism and Fertility: A Means to Different Ends?. Int. J. Mol. Sci..

[34] Hileman S.M., Tornøe J., Flier J.S., Bjørbaek C. (2000). Transcellular transport of leptin by the short leptin receptor isoform ObRa in Madin-Darby Canine Kidney cells. Endocrinology.

[35] Biernat W., Szczęsna M., Kirsz K., Zieba D.A. (2021). Seasonal and Nutritional Fluctuations in the mRNA Levels of the Short Form of the Leptin Receptor (LRa) in the Hypothalamus and Anterior Pituitary in Resistin-Treated Sheep. Animals.

[36] Münzberg H. (2010). Leptin-signaling pathways and leptin resistance. Forum Nutr..

[37] Childs G.V., Odle A.K., MacNicol M.C., MacNicol A.M. (2021). The Importance of Leptin to Reproduction. Endocrinology.

[38] Seoane-Collazo P., Martínez-Sánchez N., Milbank E., Contreras C. (2020). Incendiary Leptin. Nutrients.

[39] Villanueva E.C., Myers M.G. (2008). Leptin receptor signaling and the regulation of mammalian physiology. Int. J. Obes..

[40] Socol C.T., Chira A., Martinez-Sanchez M.A., Nuñez-Sanchez M.A., Maerescu C.M., Mierlita D., Rusu A.V., Ruiz-Alcaraz A.J., Trif M., Ramos-Molina B. (2022). Leptin Signaling in Obesity and Colorectal Cancer. Int. J. Mol. Sci..

[41] Li S., Li X. (2016). Leptin in normal physiology and leptin resistance. Sci. Bull..

[42] Quennell J.H., Mulligan A.C., Tups A., Liu X., Phipps S.J., Kemp C.J., Herbison A.E., Grattan D.R., Anderson G.M. (2009). Leptin indirectly regulates gonadotropin-releasing hormone neuronal function. Endocrinology.

[43] Stamou M.I., Georgopoulos N.A. (2018). Kallmann syndrome: Phenotype and genotype of hypogonadotropic hypogonadism. Metabolism.

[44] Cravo R.M., Margatho L.O., Osborne-Lawrence S., Donato J., Atkin S., Bookout A.L., Rovinsky S., Frazão R., Lee C.E., Gautron L. (2011). Characterization of Kiss1 neurons using transgenic mouse models. Neuroscience.

[45] Donato J., Cravo R.M., Frazão R., Gautron L., Scott M.M., Lachey J., Castro I.A., Margatho L.O., Lee S., Lee C. (2011). Leptin’s effect on puberty in mice is relayed by the ventral premammillary nucleus and does not require signaling in Kiss1 neurons. J. Clin. Investig..

[46] Petrine JC P., Franci C.R., Del Bianco-Borges B. (2020). Leptin actions through the nitrergic system to modulate the hypothalamic expression of the kiss1 mRNA in the female rat. Brain Res..

[47] Akhter N., CarlLee T., Syed M.M., Odle A.K., Cozart M.A., Haney A.C., Allensworth-James M.L., Beneš H., Childs G.V. (2014). Selective deletion of leptin receptors in gonadotropes reveals activin and GnRH-binding sites as leptin targets in support of fertility. Endocrinology.

[48] Odle A.K., Akhter N., Syed M.M., Allensworth-James M.L., Beneš H., Melgar Castillo A.I., MacNicol M.C., MacNicol A.M., Childs G.V. (2017). Leptin Regulation of Gonadotrope Gonadotropin-Releasing Hormone Receptors As a Metabolic Checkpoint and Gateway to Reproductive Competence. Front. Endocrinol..

[49] Cella F., Giordano G., Cordera R. (2000). Serum leptin concentrations during the menstrual cycle in normal-weight women: Effects of an oral triphasic estrogen-progestin medication. Eur. J. Endocrinol..

[50] Ahrens K., Mumford S.L., Schliep K.C., Kissell K.A., Perkins N.J., Wactawski-Wende J., Schisterman E.F. (2014). Serum leptin levels and reproductive function during the menstrual cycle. Am. J. Obstet. Gynecol..

[51] De Biasi S.N., Apfelbaum L.I., Apfelbaum M.E. (2001). In vitro effect of leptin on LH release by anterior pituitary glands from female rats at the time of spontaneous and steroid-induced LH surge. Eur. J. Endocrinol..

[52] Watanobe H., Schiöth H.B. (2001). Nitric oxide mediates leptin-induced preovulatory luteinizing hormone and prolactin surges in rats. Brain Res..

[53] Tipsmark C.K., Strom C.N., Bailey S.T., Borski R.J. (2008). Leptin stimulates pituitary prolactin release through an extracellular signal-regulated kinase-dependent pathway. J. Endocrinol..

[54] Kosior-Korzecka U., Bobowiec R. (2006). Leptin effect on nitric oxide and GnRH-induced FSH secretion from ovine pituitary cells in vitro. J. Physiol. Pharmacol..

[55] Richards J.S., Pangas S.A. (2010). New insights into ovarian function. Fertility Control.

[56] Salem A.M. (2021). Variation of Leptin during Menstrual Cycle and Its Relation to the Hypothalamic-Pituitary-Gonadal (HPG) Axis: A Systematic Review. Int. J. Women’s Health.

[57] Macedo T.J.S., Santos J.M.S., Bezerra M.É.S., Menezes V.G., Gouveia B.B., Barbosa L.M.R., Lins T.L.B.G., Monte A.P.O., Barberino R.S., Batista A.M. (2019). Immunolocalization of leptin and its receptor in the sheep ovary and in vitro effect of leptin on follicular development and oocyte maturation. Mol. Cell. Endocrinol..

[58] Hong K.J., Lin J.J., Lin L.H., Lai T.H. (2022). The intrafollicular concentration of leptin as a potential biomarker to predict oocyte maturity in in-vitro fertilization. Sci. Rep..

[59] Al-Aqbi M., Hart R., Ajuogu P., de Touw T.V., McFarlane J., Smart N. (2022). Follicular fluid leptin as a marker for pregnancy outcomes in women undergoing IVF treatment: A systematic review and meta-analysis. Hum. Fertil..

[60] Chou S.H., Mantzoros C. (2014). 20 years of leptin: Role of leptin in human reproductive disorders. J. Endocrinol..

[61] Cnop M., Havel P.J., Utzschneider K.M., Carr D.B., Sinha M.K., Boyko E.J., Retzlaff B.M., Knopp R.H., Brunzell J.D., Kahn S.E. (2003). Relationship of adiponectin to body fat distribution, insulin sensitivity and plasma lipoproteins: Evidence for independent roles of age and sex. Diabetologia.

[62] Ahl S., Guenther M., Zhao S., James R., Marks J., Szabo A., Kidambi S. (2015). Adiponectin Levels Differentiate Metabolically Healthy vs Unhealthy Among Obese and Nonobese White Individuals. J. Clin. Endocrinol. Metab..

[63] Nguyen T.M.D. (2020). Adiponectin: Role in Physiology and Pathophysiology. Int. J. Prev. Med..

[64] Choi H.M., Doss H.M., Kim K.S. (2020). Multifaceted Physiological Roles of Adiponectin in Inflammation and Diseases. Int. J. Mol. Sci..

[65] Kim J.E., Kim J.S., Jo M.J., Cho E., Ahn S.Y., Kwon Y.J., Ko G.J. (2022). The Roles and Associated Mechanisms of Adipokines in Development of Metabolic Syndrome. Molecules.

[66] Rak A., Mellouk N., Froment P., Dupont J. (2017). Adiponectin and resistin: Potential metabolic signals affecting hypothalamo-pituitary gonadal axis in females and males of different species. Reproduction.

[67] Hopkins T.A., Ouchi N., Shibata R., Walsh K. (2007). Adiponectin actions in the cardiovascular system. Cardiovasc. Res..

[68] Kershaw E.E., Flier J.S. (2004). Adipose tissue as an endocrine organ. J. Clin. Endocrinol. Metab..

[69] Tsatsanis C., Zacharioudaki V., Androulidaki A., Dermitzaki E., Charalampopoulos I., Minas V., Gravanis A., Margioris A.N. (2005). Adiponectin induces TNF-alpha and IL-6 in macrophages and promotes tolerance to itself and other pro-inflammatory stimuli. Biochem. Biophys. Res. Commun..

[70] Fang H., Judd R.L. (2018). Adiponectin Regulation and Function. Compr. Physiol..

[71] Haugen F., Drevon C.A. (2007). Activation of nuclear factor-kappaB by high molecular weight and globular adiponectin. Endocrinology.

[72] Yau S.Y., Li A., Hoo R.L., Ching Y.P., Christie B., Lee T.M., Xu A., So K.F. (2014). Physical exercise-induced hippocampal neurogenesis and antidepressant effects are mediated by the adipocyte hormone adiponectin. Proc. Natl. Acad. Sci. USA.

[73] Wang Z.V., Scherer P.E. (2016). Adiponectin, the past two decades. J. Mol. Cell Biol..

[74] Sun J., Gao Y., Yao T., Huang Y., He Z., Kong X., Yu K.J., Wang R.T., Guo H., Yan J. (2016). Adiponectin potentiates the acute effects of leptin in arcuate *Pomc neurons*. Mol. Metab..

[75] Lee T.H., Cheng K.K., Hoo R.L., Siu P.M., Yau S.Y. (2019). The Novel Perspectives of Adipokines on Brain Health. Int. J. Mol. Sci..

[76] Wen J.P., Lv W.S., Yang J., Nie A.F., Cheng X.B., Yang Y., Ge Y., Li X.Y., Ning G. (2008). Globular adiponectin inhibits GnRH secretion from GT1-7 hypothalamic GnRH neurons by induction of hyperpolarization of membrane potential. Biochem. Biophys. Res. Commun..

[77] Wen J.P., Liu C., Bi W.K., Hu Y.T., Chen Q., Huang H., Liang J.X., Li L.T., Lin L.X., Chen G. (2012). Adiponectin inhibits KISS1 gene transcription through AMPK and specificity protein-1 in the hypothalamic GT1-7 neurons. J. Endocrinol..

[78] Estienne A., Bongrani A., Reverchon M., Ramé C., Ducluzeau P.H., Froment P., Dupont J. (2019). Involvement of Novel Adipokines, Chemerin, Visfatin, Resistin and Apelin in Reproductive Functions in Normal and Pathological Conditions in Humans and Animal Models. Int. J. Mol. Sci..

[79] Fazeli P.K., Bredella M.A., Pachon-Peña G., Zhao W., Zhang X., Faje A.T., Resulaj M., Polineni S.P., Holmes T.M., Lee H. (2021). The dynamics of human bone marrow adipose tissue in response to feeding and fasting. JCI Insight.

[80] Patel L., Buckels A.C., Kinghorn I.J., Murdock P.R., Holbrook J.D., Plumpton C., Macphee C.H., Smith S.A. (2003). Resistin is expressed in human macrophages and directly regulated by PPAR gamma activators. Biochem. Biophys. Res. Commun..

[81] Pestel J., Blangero F., Watson J., Pirola L., Eljaafari A. (2023). Adipokines in obesity and metabolic-related-diseases. Biochimie.

[82] Kaminska B., Kurowicka B., Kiezun M., Dobrzyn K., Kisielewska K., Gudelska M., Kopij G., Szymanska K., Zarzecka B., Koker O. (2024). The Role of Adipokines in the Control of Pituitary Functions. Animals.

[83] Flores-Cordero J.A., Pérez-Pérez A., Jiménez-Cortegana C., Alba G., Flores-Barragán A., Sánchez-Margalet V. (2022). Obesity as a Risk Factor for Dementia and Alzheimer’s Disease: The Role of Leptin. Int. J. Mol. Sci..

[84] Morash B.A., Ur E., Wiesner G., Roy J., Wilkinson M. (2004). Pituitary resistin gene expression: Effects of age, gender and obesity. Neuroendocrinology.

[85] Maillard V., Elis S., Desmarchais A., Hivelin C., Lardic L., Lomet D., Uzbekova S., Monget P., Dupont J. (2017). Visfatin and resistin in gonadotroph cells: Expression, regulation of LH secretion and signalling pathways. Reprod. Fertil. Dev..

[86] Acquarone E., Monacelli F., Borghi R., Nencioni A., Odetti P. (2019). Resistin: A reappraisal. Mech. Ageing Dev..

[87] Rak-Mardyła A., Durak M., Lucja Gregoraszczuk E. (2013). Effects of resistin on porcine ovarian follicle steroidogenesis in prepubertal animals: An in vitro study. Reprod. Biol. Endocrinol..

[88] Reverchon M., Cornuau M., Ramé C., Guerif F., Royère D., Dupont J. (2013). Resistin decreases insulin-like growth factor I-induced steroid production and insulin-like growth factor I receptor signaling in human granulosa cells. Fertil. Steril..

[89] Nikanfar S., Oghbaei H., Rastgar Rezaei Y., Zarezadeh R., Jafari-Gharabaghlou D., Nejabati H.R., Bahrami Z., Bleisinger N., Samadi N., Fattahi A. (2021). Role of adipokines in the ovarian function: Oogenesis and steroidogenesis. J. Steroid Biochem. Mol. Biol..

[90] Yoshino J., Mills K.F., Yoon M.J., Imai S. (2011). Nicotinamide mononucleotide, a key NAD(+) intermediate, treats the pathophysiology of diet- and age-induced diabetes in mice. Cell Metab..

[91] Higgins C.B., Mayer A.L., Zhang Y., Franczyk M., Ballentine S., Yoshino J., DeBosch B.J. (2022). SIRT1 selectively exerts the metabolic protective effects of hepatocyte nicotinamide phosphoribosyltransferase. Nat. Commun..

[92] Nielsen K.N., Peics J., Ma T., Karavaeva I., Dall M., Chubanava S., Basse A.L., Dmytriyeva O., Treebak J.T., Gerhart-Hines Z. (2018). NAMPT-mediated NAD(+) biosynthesis is indispensable for adipose tissue plasticity and development of obesity. Mol. Metab..

[93] Mlyczyńska E., Kieżun M., Kurowska P., Dawid M., Pich K., Respekta N., Daudon M., Rytelewska E., Dobrzyń K., Kamińska B. (2022). New Aspects of Corpus Luteum Regulation in Physiological and Pathological Conditions: Involvement of Adipokines and Neuropeptides. Cells.

[94] Saddi-Rosa P., Oliveira C.S., Giuffrida F.M., Reis A.F. (2010). Visfatin, glucose metabolism and vascular disease: A review of evidence. Diabetol. Metab. Syndr..

[95] Heo Y.J., Choi S.E., Jeon J.Y., Han S.J., Kim D.J., Kang Y., Lee K.W., Kim H.J. (2019). Visfatin Induces Inflammation and Insulin Resistance via the NF-κB and STAT3 Signaling Pathways in Hepatocytes. J. Diabetes Res..

[96] Zorena K., Jachimowicz-Duda O., Ślęzak D., Robakowska M., Mrugacz M. (2020). Adipokines and Obesity. Potential Link to Metabolic Disorders and Chronic Complications. Int. J. Mol. Sci..

[97] Li Z., Wang Y., Tian X., Shang P., Chen H., Kang X., Tian Y., Han R. (2017). Characterization of the visfatin gene and its expression pattern and effect on 3T3-L1 adipocyte differentiation in chickens. Gene.

[98] Dahl T.B., Holm S., Aukrust P., Halvorsen B. (2012). Visfatin/NAMPT: A multifaceted molecule with diverse roles in physiology and pathophysiology. Annu. Rev. Nutr..

[99] Jacques C., Holzenberger M., Mladenovic Z., Salvat C., Pecchi E., Berenbaum F., Gosset M. (2012). Proinflammatory actions of visfatin/nicotinamide phosphoribosyltransferase (Nampt) involve regulation of insulin signaling pathway and Nampt enzymatic activity. J. Biol. Chem..

[100] Romacho T., Villalobos L.A., Cercas E., Carraro R., Sánchez-Ferrer C.F., Peiró C. (2013). Visfatin as a novel mediator released by inflamed human endothelial cells. PLoS ONE.

[101] Kim S.R., Bae Y.H., Bae S.K., Choi K.S., Yoon K.H., Koo T.H., Jang H.O., Yun I., Kim K.W., Kwon Y.G. (2008). Visfatin enhances ICAM-1 and VCAM-1 expression through ROS-dependent NF-kappaB activation in endothelial cells. Biochim. Biophys. Acta.

[102] Agbaedeng T.A., Iroga P.E., Rathnasekara V.M., Zacharia A.L. (2024). Adipokines and stroke: A systematic review and meta-analysis of disease risk and patient outcomes. Obes. Rev..

[103] Yang Y., Li Z., Tao H.F., Qi X.Y., Wang W.L., Yang L., Wang H., Xu P. (2014). An elevated plasma level of visfatin increases the risk of myocardial infarction. Genet. Mol. Res..

[104] Chang Y.H., Chang D.M., Lin K.C., Shin S.J., Lee Y.J. (2011). Visfatin in overweight/obesity, type 2 diabetes mellitus, insulin resistance, metabolic syndrome and cardiovascular diseases: A meta-analysis and systemic review. Diabetes Metab. Res. Rev..

[105] Kamińska A., Kopczyńska E., Bronisz A., Zmudzińska M., Bieliński M., Borkowska A., Tyrakowski T., Junik R. (2010). An evaluation of visfatin levels in obese subjects. Endokrynol. Pol..

[106] Chen J., Hu L., Ling Y., Xu Y., Yu X., Ma L., Shou M. (2023). Risk Correlation Analysis between Polycystic Ovary Syndrome (PCOS) and Serum Visfatin Levels in Middle-Aged Women: Systematic Review and Meta-Analysis. Discov. Med..

[107] Lin K., Sun X., Wang X., Wang H., Chen X. (2020). Circulating Adipokine Levels in Nonobese Women with Polycystic Ovary Syndrome and in Nonobese Control Women: A Systematic Review and Meta-Analysis. Front. Endocrinol..

[108] Szymanska K., Zaobidna E., Rytelewska E., Mlyczynska E., Kurowska P., Dobrzyn K., Kiezun M., Kaminska B., Smolinska N., Rak A. (2023). Visfatin in the porcine pituitary gland: Expression and regulation of secretion during the oestrous cycle and early pregnancy. Sci. Rep..

[109] Kurowska P., Mlyczyńska E., Dawid M., Sierpowski M., Estienne A., Dupont J., Rak A. (2021). Adipokines change the balance of proliferation/apoptosis in the ovarian cells of human and domestic animals: A comparative review. Anim. Reprod. Sci..

[110] Kaminski T., Kiezun M., Zaobidna E., Dobrzyn K., Wasilewska B., Mlyczynska E., Rytelewska E., Kisielewska K., Gudelska M., Bors K. (2021). Plasma level and expression of visfatin in the porcine hypothalamus during the estrous cycle and early pregnancy. Sci. Rep..

[111] Hallschmid M., Randeva H., Tan B.K., Kern W., Lehnert H. (2009). Relationship between cerebrospinal fluid visfatin (PBEF/Nampt) levels and adiposity in humans. Diabetes.

[112] Reverchon M., Cornuau M., Cloix L., Ramé C., Guerif F., Royère D., Dupont J. (2013). Visfatin is expressed in human granulosa cells: Regulation by metformin through AMPK/SIRT1 pathways and its role in steroidogenesis. Mol. Hum. Reprod..

[113] Estienne A., Brossaud A., Reverchon M., Ramé C., Froment P., Dupont J. (2020). Adipokines Expression and Effects in Oocyte Maturation, Fertilization and Early Embryo Development: Lessons from Mammals and Birds. Int. J. Mol. Sci..

[114] Reverchon M., Rame C., Bunel A., Chen W., Froment P., Dupont J. (2016). VISFATIN (NAMPT) Improves In Vitro IGF1-Induced Steroidogenesis and IGF1 Receptor Signaling Through SIRT1 in Bovine Granulosa Cells. Biol. Reprod..

[115] Kim J.A., Choi K.M. (2020). Newly Discovered Adipokines: Pathophysiological Link between Obesity and Cardiometabolic Disorders. Front. Physiol..

[116] Dupont J., Pollet-Villard X., Reverchon M., Mellouk N., Levy R. (2015). Adipokines in human reproduction. Horm. Mol. Biol. Clin. Investig..

[117] Cătoi A.F., Suciu Ş., Pârvu A.E., Copăescu C., Galea R.F., Buzoianu A.D., Vereşiu I.A., Cătoi C., Pop I.D. (2014). Increased chemerin and decreased omentin-1 levels in morbidly obese patients are correlated with insulin resistance, oxidative stress and chronic inflammation. Clujul Med..

[118] Ismail S.A., Mohamed S.A. (2012). Serum levels of visfatin and omentin-1 in patients with psoriasis and their relation to disease severity. Br. J. Dermatol..

[119] Yin J., Hou P., Wu Z., Nie Y. (2015). Decreased levels of serum omentin-1 in patients with inflammatory bowel disease. Med. Sci. Monit..

[120] Yang R.Z., Lee M.J., Hu H., Pray J., Wu H.B., Hansen B.C., Shuldiner A.R., Fried S.K., McLenithan J.C., Gong D.W. (2006). Identification of omentin as a novel depot-specific adipokine in human adipose tissue: Possible role in modulating insulin action. Am. J. Physiol. Endocrinol. Metab..

[121] Zhou J.Y., Chan L., Zhou S.W. (2014). Omentin: Linking metabolic syndrome and cardiovascular disease. Curr. Vasc. Pharmacol..

[122] Watanabe K., Watanabe R., Konii H., Shirai R., Sato K., Matsuyama T.A., Ishibashi-Ueda H., Koba S., Kobayashi Y., Hirano T. (2016). Counteractive effects of omentin-1 against atherogenesis†. Cardiovasc. Res..

[123] Lin X., Sun Y., Yang S., Yu M., Pan L., Yang J., Yang J., Shao Q., Liu J., Liu Y. (2021). Omentin-1 Modulates Macrophage Function via Integrin Receptors αvβ3 and αvβ5 and Reverses Plaque Vulnerability in Animal Models of Atherosclerosis. Front. Cardiovasc. Med..

[124] Leandro A., Queiroz M., Azul L., Seiça R., Sena C.M. (2021). Omentin: A novel therapeutic approach for the treatment of endothelial dysfunction in type 2 diabetes. Free Radic. Biol. Med..

[125] Zhong X., Li X., Liu F., Tan H., Shang D. (2012). Omentin inhibits TNF-α-induced expression of adhesion molecules in endothelial cells via ERK/NF-κB pathway. Biochem. Biophys. Res. Commun..

[126] Yamawaki H., Kuramoto J., Kameshima S., Usui T., Okada M., Hara Y. (2011). Omentin, a novel adipocytokine inhibits TNF-induced vascular inflammation in human endothelial cells. Biochem. Biophys. Res. Commun..

[127] Brunetti L., Orlando G., Ferrante C., Recinella L., Leone S., Chiavaroli A., Di Nisio C., Shohreh R., Manippa F., Ricciuti A. (2013). Orexigenic effects of omentin-1 related to decreased CART and CRH gene expression and increased norepinephrine synthesis and release in the hypothalamus. Peptides.

[128] Respekta N., Pich K., Mlyczyńska E., Dobrzyń K., Ramé C., Kamiński T., Smolińska N., Dupont J., Rak A. (2023). Plasma level of omentin-1, its expression, and its regulation by gonadotropin-releasing hormone and gonadotropins in porcine anterior pituitary cells. Sci. Rep..

[129] Schäffler A., Neumeier M., Herfarth H., Fürst A., Schölmerich J., Büchler C. (2005). Genomic structure of human omentin, a new adipocytokine expressed in omental adipose tissue. Biochim. Biophys. Acta.

[130] Cloix L., Reverchon M., Cornuau M., Froment P., Ramé C., Costa C., Froment G., Lecomte P., Chen W., Royère D. (2014). Expression and Regulation of INTELECTIN1 in Human Granulosa-Lutein Cells: Role in IGF-1-Induced Steroidogenesis Through NAMPT1. Biol. Reprod..

[131] Sirotkin A.V., Fabová Z., Loncová B., Bauerová M., Halim Harrath A. (2024). The adipokines progranulin and omentin—Novel regulators of basic ovarian cell functions. Reprod. Biol. Endocrinol..

[132] Tang Y.L., Yu J., Zeng Z.G., Liu Y., Liu J.Y., Xu J.X. (2017). Circulating omentin-1 levels in women with polycystic ovary syndrome: A meta-analysis. Gynecol. Endocrinol..

[133] Mahde A., Shaker M., Al-Mashhadani Z. (2009). Study of Omentin1 and Other Adipokines and Hormones in PCOS Patients. Oman Med. J..

[134] Shaker M., Mashhadani Z.I., Mehdi A.A. (2010). Effect of Treatment with Metformin on Omentin-1, Ghrelin and other Biochemical, Clinical Features in PCOS Patients. Oman Med. J..

[135] Tan B.K., Adya R., Farhatullah S., Chen J., Lehnert H., Randeva H.S. (2010). Metformin treatment may increase omentin-1 levels in women with polycystic ovary syndrome. Diabetes.

[136] Yan P., Li L., Yang M., Liu D., Liu H., Boden G., Yang G. (2011). Effects of the long-acting human glucagon-like peptide-1 analog liraglutide on plasma omentin-1 levels in patients with type 2 diabetes mellitus. Diabetes Res. Clin. Pract..

[137] Franik G., Sadlocha M., Madej P., Owczarek A., Skrzypulec-Plinta V., Plinta R., Chudek J., Olszanecka-Glinianowicz M. (2020). Circulating omentin-1 levels and inflammation in polycystic ovary syndrome. Ginekol. Pol..

[138] Rudnicka E., Suchta K., Grymowicz M., Calik-Ksepka A., Smolarczyk K., Duszewska A.M., Smolarczyk R., Meczekalski B. (2021). Chronic Low Grade Inflammation in Pathogenesis of PCOS. Int. J. Mol. Sci..

[139] Villa P.A., Ruggiero-Ruff R.E., Jamieson B.B., Campbell R.E., Coss D. (2024). Obesity alters POMC and kisspeptin neuron crosstalk leading to reduced luteinizing hormone in male mice. J. Neurosci..

[140] Gao M., Tao X., Zhang Q., He W., Zhao T., Yuan T. (2023). Correlation between kisspeptin and biochemical markers in obese and non-obese women with polycystic ovary syndrome. Gynecol. Endocrinol..

[141] Ohtaki T., Shintani Y., Honda S., Matsumoto H., Hori A., Kanehashi K., Terao Y., Kumano S., Takatsu Y., Masuda Y. (2001). Metastasis suppressor gene KiSS-1 encodes peptide ligand of a G-protein-coupled receptor. Nature.

[142] Kotani M., Detheux M., Vandenbogaerde A., Communi D., Vanderwinden J.M., Le Poul E., Brézillon S., Tyldesley R., Suarez-Huerta N., Vandeput F. (2001). The metastasis suppressor gene KiSS-1 encodes kisspeptins, the natural ligands of the orphan G protein-coupled receptor GPR54. J. Biol. Chem..

[143] Manfredi-Lozano M., Roa J., Ruiz-Pino F., Piet R., Garcia-Galiano D., Pineda R., Zamora A., Leon S., Sanchez-Garrido M.A., Romero-Ruiz A. (2016). Defining a novel leptin-melanocortin-kisspeptin pathway involved in the metabolic control of puberty. Mol. Metab..

[144] Avendaño M.S., Vazquez M.J., Tena-Sempere M. (2017). Disentangling puberty: Novel neuroendocrine pathways and mechanisms for the control of mammalian puberty. Hum. Reprod. Update.

[145] Navarro V.M. (2020). Metabolic regulation of kisspeptin—The link between energy balance and reproduction. Nat. Rev. Endocrinol..

[146] Lehman M.N., Coolen L.M., Goodman R.L. (2010). Minireview: Kisspeptin/neurokinin B/dynorphin (KNDy) cells of the arcuate nucleus: A central node in the control of gonadotropin-releasing hormone secretion. Endocrinology.

[147] Xie Q., Kang Y., Zhang C., Xie Y., Wang C., Liu J., Yu C., Zhao H., Huang D. (2022). The Role of Kisspeptin in the Control of the Hypothalamic-Pituitary-Gonadal Axis and Reproduction. Front. Endocrinol..

[148] Zhang C., Bosch M.A., Qiu J., Rønnekleiv O.K., Kelly M.J. (2015). 17β-Estradiol increases persistent Na(+) current and excitability of AVPV/PeN Kiss1 neurons in female mice. Mol. Endocrinol..

[149] Cheng G., Coolen L.M., Padmanabhan V., Goodman R.L., Lehman M.N. (2010). The kisspeptin/neurokinin B/dynorphin (KNDy) cell population of the arcuate nucleus: Sex differences and effects of prenatal testosterone in sheep. Endocrinology.

[150] Novaira H.J., Sonko M.L., Radovick S. (2016). Kisspeptin Induces Dynamic Chromatin Modifications to Control GnRH Gene Expression. Mol. Neurobiol..

[151] Abbara A., Eng P.C., Phylactou M., Clarke S.A., Richardson R., Sykes C.M., Phumsatitpong C., Mills E., Modi M., Izzi-Engbeaya C. (2020). Kisspeptin receptor agonist has therapeutic potential for female reproductive disorders. J. Clin. Investig..

[152] Abbara A., Ufer M., Voors-Pette C., Berman L., Ezzati M., Wu R., Lee T.Y., Ferreira J.C.A., Migoya E., Dhillo W.S. (2024). Endocrine profile of the kisspeptin receptor agonist MVT-602 in healthy premenopausal women with and without ovarian stimulation: Results from 2 randomized, placebo-controlled clinical tricals. Fertil. Steril..

[153] Cejudo Roman A., Pinto F.M., Dorta I., Almeida T.A., Hernández M., Illanes M., Tena-Sempere M., Candenas L. (2012). Analysis of the expression of neurokinin B, kisspeptin, and their cognate receptors NK3R and KISS1R in the human female genital tract. Fertil. Steril..

[154] Owens L.A., Abbara A., Lerner A., O’floinn S., Christopoulos G., Khanjani S., Islam R., Hardy K., Hanyaloglu A.C., Lavery S.A. (2018). The direct and indirect effects of kisspeptin-54 on granulosa lutein cell function. Hum. Reprod..

[155] Chang B., Song C., Gao H., Ma T., Li T., Ma Q., Yao T., Wang M., Li J., Yi X. (2021). Leptin and inflammatory factors play a synergistic role in the regulation of reproduction in male mice through hypothalamic kisspeptin-mediated energy balance. Reprod. Biol. Endocrinol..

[156] Iwasa T., Matsuzaki T., Tungalagsuvd A., Munkhzaya M., Kawami T., Niki H., Kato T., Kuwahara A., Uemura H., Yasui T. (2014). Hypothalamic Kiss1 and RFRP gene expressions are changed by a high dose of lipopolysaccharide in female rats. Horm. Behav..

[157] Sarchielli E., Comeglio P., Squecco R., Ballerini L., Mello T., Guarnieri G., Idrizaj E., Mazzanti B., Vignozzi L., Gallina P. (2017). Tumor Necrosis Factor-α Impairs Kisspeptin Signaling in Human Gonadotropin-Releasing Hormone Primary Neurons. J. Clin. Endocrinol. Metab..

[158] Wahab F., Bano R., Jabeen S., Irfan S., Shahab M. (2010). Effect of peripheral kisspeptin administration on adiponectin, leptin, and resistin secretion under fed and fasting conditions in the adult male rhesus monkey (*Macaca mulatta*). Horm. Metab. Res..

[159] Negi N., Das B.K. (2018). CNS: Not an immunoprivilaged site anymore but a virtual secondary lymphoid organ. Int. Rev. Immunol..

[160] Léon S., Nadjar A., Quarta C. (2021). Microglia-Neuron Crosstalk in Obesity: Melodious Interaction or Kiss of Death?. Int. J. Mol. Sci..

[161] Boleti A.P.D.A., de O Cardoso P.H., Frihling B.E., E Silva P.S., de Moraes L.F.R.N., Migliolo L. (2023). Adipose tissue, systematic inflammation, and neurodegenerative diseases. Neural Regen. Res..

[162] Valdearcos M., Myers M.G., Koliwad S.K. (2019). Hypothalamic microglia as potential regulators of metabolic physiology. Nat. Metab..

[163] Dorfman M.D., Thaler J.P. (2015). Hypothalamic inflammation and gliosis in obesity. Curr. Opin. Endocrinol. Diabetes Obes..

[164] Paolicelli R.C., Bisht K., Tremblay M. (2014). Fractalkine regulation of microglial physiology and consequences on the brain and behavior. Front. Cell. Neurosci..

[165] Dorfman M.D., Krull J.E., Douglass J.D., Fasnacht R., Lara-Lince F., Meek T.H., Shi X., Damian V., Nguyen H.T., Matsen M.E. (2017). Sex differences in microglial CX3CR1 signalling determine obesity susceptibility in mice. Nat. Commun..

[166] Chen K.E., Lainez N.M., Nair M.G., Coss D. (2021). Visceral adipose tissue imparts peripheral macrophage influx into the hypothalamus. J. Neuroinflamm..

[167] Lainez N.M., Jonak C.R., Nair M.G., Ethell I.M., Wilson E.H., Carson M.J., Coss D. (2018). Diet-Induced Obesity Elicits Macrophage Infiltration and Reduction in Spine Density in the Hypothalami of Male but Not Female Mice. Front. Immunol..

[168] Chang E.H., Chavan S.S., Pavlov V.A. (2019). Cholinergic Control of Inflammation, Metabolic Dysfunction, and Cognitive Impairment in Obesity-Associated Disorders: Mechanisms and Novel Therapeutic Opportunities. Front. Neurosci..

[169] Barabás K., Szabó-Meleg E., Ábrahám I.M. (2020). Effect of Inflammation on Female Gonadotropin-Releasing Hormone (GnRH) Neurons: Mechanisms and Consequences. Int. J. Mol. Sci..

[170] Wojtulewicz K., Krawczyńska A., Tomaszewska-Zaremba D., Wójcik M., Herman A.P. (2020). Effect of Acute and Prolonged Inflammation on the Gene Expression of Proinflammatory Cytokines and Their Receptors in the Anterior Pituitary Gland of Ewes. Int. J. Mol. Sci..

[171] Navarro G., Allard C., Xu W., Mauvais-Jarvis F. (2015). The role of androgens in metabolism, obesity, and diabetes in males and females. Obesity.

[172] Lonardo M.S., Cacciapuoti N., Guida B., Di Lorenzo M., Chiurazzi M., Damiano S., Menale C. (2024). Hypothalamic-Ovarian axis and Adiposity Relationship in Polycystic Ovary Syndrome: Physiopathology and Therapeutic Options for the Management of Metabolic and Inflammatory Aspects. Curr. Obes. Rep..

[173] Biernacka-Bartnik A., Kocełak P., Owczarek A.J., Choręza P., Puzianowska-Kuźnicka M., Markuszewski L., Madej P., Chudek J., Olszanecka-Glinianowicz M. (2022). Prediction of Insulin Resistance and Impaired Fasting Glucose Based on Sex Hormone-Binding Globulin (SHBG) Levels in Polycystic Ovary Syndrome. Int. J. Endocrinol..

[174] Rosenfield R.L., Ehrmann D.A. (2016). The Pathogenesis of Polycystic Ovary Syndrome (PCOS): The Hypothesis of PCOS as Functional Ovarian Hyperandrogenism Revisited. Endocr. Rev..

[175] Malini N.A., Roy G.K. (2021). Influence of Insulin on LH, Testosterone and SHBG in various PCOS Categories based on the Mode of Secretion of LH in relation to FSH Levels. Acta Endocrinol..

[176] Das D., Arur S. (2017). Conserved insulin signaling in the regulation of oocyte growth, development, and maturation. Mol. Reprod. Dev..

[177] Sakumoto T., Tokunaga Y., Tanaka H., Nohara M., Motegi E., Shinkawa T., Nakaza A., Higashi M. (2010). Insulin resistance/hyperinsulinemia and reproductive disorders in infertile women. Reprod. Med. Biol..

[178] Tay C.T., Garrad R., Mousa A., Bahri M., Joham A., Teede H. (2023). Polycystic ovary syndrome (PCOS): International collaboration to translate evidence and guide future research. J. Endocrinol..

[179] Teede H.J., Tay C.T., Laven J.J.E., Dokras A., Moran L.J., Piltonen T.T., Costello M.F., Boivin J., Redman L.M., Boyle J.A. (2023). Recommendations from the 2023 international evidence-based guideline for the assessment and management of polycystic ovary syndrome. Eur. J. Endocrinol..

[180] Garad R., Shorakae S., Teede H. (2019). Assessment and management of women with polycystic ovary syndrome (PCOS). Advanced Practice in Endocrinology Nursing.

[181] Aggarwal M., Chakole S. (2023). Prevalence of Polycystic Ovarian Syndrome and Its Link to Obesity in Adolescent Girls. Cureus.

[182] Liu Q., Zhu Z., Kraft P., Deng Q., Stener-Victorin E., Jiang X. (2022). Genomic correlation, shared loci, and causal relationship between obesity and polycystic ovary syndrome: A large-scale genome-wide cross-trait analysis. BMC Med..

[183] Barrea L., Muscogiuri G., Pugliese G., de Alteriis G., Colao A., Savastano S. (2021). Metabolically Healthy Obesity (MHO) vs. Metabolically Unhealthy Obesity (MUO) Phenotypes in PCOS: Association with Endocrine-Metabolic Profile, Adherence to the Mediterranean Diet, and Body Composition. Nutrients.

[184] Zhang Y., Cai M., Dilimulati D., Lin Z., Sun H., Cui R., Fei H., Gao X., Zeng Q., Shao X. (2021). Correlation between Serum Uric Acid and Body Fat Distribution in Patients with Polycystic Ovary Syndrome. Front. Endocrinol..

[185] Yildiz B.O., Bozdag G., Yapici Z., Esinler I., Yarali H. (2012). Prevalence, phenotype and cardiometabolic risk of polycystic ovary syndrome under different diagnostic criteria. Hum. Reprod..

[186] Alvarez-Blasco F., Botella-Carretero J.I., San Millán J.L., Escobar-Morreale H.F. (2006). Prevalence and characteristics of the polycystic ovary syndrome in overweight and obese women. Arch. Intern. Med..

[187] Walters K.A., Allan C.M., Handelsman D.J. (2012). Rodent models for human polycystic ovary syndrome. Biol. Reprod..

[188] Caldwell A.S., Middleton L.J., Jimenez M., Desai R., McMahon A.C., Allan C.M., Handelsman D.J., Walters K.A. (2014). Characterization of reproductive, metabolic, and endocrine features of polycystic ovary syndrome in female hyperandrogenic mouse models. Endocrinology.

[189] Zhao H., Zhang J., Cheng X., Nie X., He B. (2023). Insulin resistance in polycystic ovary syndrome across various tissues: An updated review of pathogenesis, evaluation, and treatment. J. Ovarian Res..

[190] Chang H.M., Wu H.C., Sun Z.G., Lian F., Leung P.C.K. (2019). Neurotrophins and glial cell line-derived neurotrophic factor in the ovary: Physiological and pathophysiological implications. Hum. Reprod. Update.

[191] Urbanek M., Du Y., Silander K., Collins F.S., Steppan C.M., Strauss J.F., Dunaif A., Spielman R.S., Legro R.S. (2003). Variation in resistin gene promoter not associated with polycystic ovary syndrome. Diabetes.

[192] Pizzuti A., Argiolas A., Di Paola R., Baratta R., Rauseo A., Bozzali M., Vigneri R., Dallapiccola B., Trischitta V., Frittitta L. (2002). An ATG repeat in the 3′-untranslated region of the human resistin gene is associated with a decreased risk of insulin resistance. J. Clin. Endocrinol. Metab..

[193] Majuri A., Santaniemi M., Rautio K., Kunnari A., Vartiainen J., Ruokonen A., Kesäniemi Y.A., Tapanainen J.S., Ukkola O., Morin-Papunen L. (2007). Rosiglitazone treatment increases plasma levels of adiponectin and decreases levels of resistin in overweight women with PCOS: A randomized placebo-controlled study. Eur. J. Endocrinol..

[194] Dumesic D.A., Phan J.D., Leung K.L., Grogan T.R., Ding X., Li X., Hoyos L.R., Abbott D.H., Chazenbalk G.D. (2019). Adipose Insulin Resistance in Normal-Weight Women with Polycystic Ovary Syndrome. J. Clin. Endocrinol. Metab..

[195] Dhurandhar E.J., Keith S.W. (2014). The aetiology of obesity beyond eating more and exercising less. Best. Pract. Res. Clin. Gastroenterol..

[196] Yao S., Lopez-Tello J., Sferruzzi-Perri A.N. (2021). Developmental programming of the female reproductive system-a review. Biol. Reprod..

[197] Sobrino V., Avendaño M.S., Perdices-López C., Jimenez-Puyer M., Tena-Sempere M. (2022). Kisspeptins and the neuroendocrine control of reproduction: Recent progress and new frontiers in kisspeptin research. Front. Neuroendocrinol..

[198] Jensterle M., Janez A., Fliers E., DeVries J.H., Vrtacnik-Bokal E., Siegelaar S.E. (2019). The role of glucagon-like peptide-1 in reproduction: From physiology to therapeutic perspective. Hum. Reprod. Update.

